# Dropout from Court-Mandated Intervention Programs for Intimate Partner Violence Offenders: The Relevance of Alcohol Misuse and Cognitive Impairments

**DOI:** 10.3390/ijerph16132402

**Published:** 2019-07-06

**Authors:** Ángel Romero-Martínez, Marisol Lila, Enrique Gracia, Luis Moya-Albiol

**Affiliations:** 1 Department of Psychobiology, University of Valencia, 46010 Valencia, Spain; 2 Department of Social Psychology, University of Valencia, 46010 Valencia, Spain

**Keywords:** alcohol, dropout, empathy, intimate partner violence, recidivism

## Abstract

There is considerable interest in offering insight into the mechanisms that might explain why certain perpetrators of intimate partner violence against women (IPVAW) drop out of interventions. Although several socio-demographic variables and attitudes towards IPVAW have been proposed as risk factors for IPVAW perpetrators’ dropout, less attention has been paid to alcohol misuse, and its interactions with empathic and cognitive deficits, in the discontinuation of the treatment. Therefore, the main aim of this study was to compare the profile of a carefully selected sample of IPVAW perpetrators, divided into four groups: those who completed the intervention with low (*n* = 267) and high alcohol consumption (*n* = 67); and those who dropped out before the intervention ended with low (*n* = 62).and high alcohol consumption (*n* = 27). Furthermore, we also studied the initial risk before the intervention started and the official IPVAW recidivism during the first year after the intervention ended. Our results revealed that IPVAW perpetrators, especially those who did not complete the intervention and had high alcohol consumption/alcohol misuse, were less accurate in decoding emotional facial signals and presented more errors and perseverative errors than those who completed the intervention. These differences were particularly marked in comparison with those who showed less alcohol consumption. Furthermore, the first group also presented the highest risk (assessed by therapists) and official recidivism rate (official records). Conversely, the lowest rate of IPVAW reoffending was presented by IPVAW treatment completers with low alcohol consumption. Hence, our study identifies different targets that should be addressed during the initial stages of interventions in order to prevent or reduce IPVAW dropout, which in turn might reduce violence recidivism in the long term through their effects on emotional information processing and behavioural regulation.

## 1. Introduction

Intimate partner violence against women (IPVAW) is a major worldwide health problem with a lifetime prevalence of around 23% and severe consequences for women’s health [1,2]. Hence, a large number of studies have focused on the victims, but it is also important to study IPVAW offenders/perpetrators in order to understand this complex phenomenon. The greater our understanding of this phenomenon, the more effective the interventions will be.

In order to prevent and punish IPVAW, Spanish legislation passed the comprehensive Law 1/2004 to protect women from any kind of psychological, physical, and/or sexual violence perpetrated by their male partners in an intimate relationship. This law defined IPVAW as “an expression of discrimination, unequal status, and power relations of men over women, (…) by their present or former partners or by those who are or have been connected by similar relations of affectivity, even without cohabitation” [3]. Some of the strategies implemented in this law were the creation of a State Observatory on violence against women and a firm commitment to developing specialized legal services through the establishment of Violence against Women courts and the figure of a Prosecutor to deal with cases of Violence Against Women. Furthermore, Title IV of this law introduced some norms into the context of criminal law. There was an increase in the penalties for IPVAW crimes, and this law involved the need to develop batterer intervention programs (BIP) in Spain. Mainly based on cognitive-behavioural therapies (CBT), these programs are designed for men in prison and men who have been convicted of IPVAW with suspended sentences (less than 2 years in prison and no previous criminal record of IPVAW). IPVAW perpetrators with suspended sentences have to attend intervention programs and comply with a mandatory no-contact order with their victims [3,4,5,6,7,8,9].

Many studies have offered insight into the mechanisms that might explain why certain IPVAW perpetrators drop out of the intervention before it ends. In this regard, there is certain unanimity in assuming that treatment noncompliance/dropout is associated with a higher risk of reoffending [3,4,5,6,7,8,9]. Thus, it is necessary to know which main variables tend to facilitate dropout, in order to develop more effective treatment approaches. This kind of research would guide the development of coadjutant treatments and intervention packages to reinforce treatment adherence and decrease the risk of future IPVAW recidivism.

Several socio-demographic variables, such as age, educational level, marital status, employment, income, and previous criminal history, have been highlighted as important factors in discontinuing the intervention. Individuals who tend to leave the intervention are characterized by being younger, with a low educational level, single, and unemployed, and by having a low-income level and a previous criminal record [8,10,11,12,13,14]. Moreover, other studies have postulated that IPVAW perpetrators could be characterized by maintaining tolerant attitudes towards IPVAW and hostile/sexist cognitive schemas towards women [8,15,16]. Nonetheless, the majority of the studies in this field have mentioned heavy and sustained alcohol consumption/misuse as the most relevant factor in intervention success and treatment adherence, particularly associated with treatment discontinuation during its initial stages [4,5,17]. Furthermore, researchers emphasize that alcohol misuse heightens the likelihood of IPVAW reoffending [5,18], with IPVAW offenders with heavy alcohol use presenting the highest rates of dropout and IPVAW recidivism [8].

The previously mentioned studies presented a series of socio-demographic variables and attitudes towards IPVAW that are extremely important in designing intervention programs, but none of them paid attention to other variables such as empathic skills, emotion decoding abilities, and executive functioning (i.e., cognitive flexibility). All of these cognitive skills are relevant in behavioural and emotional regulation and cognitive processing [19,20,21,22,23,24]. Additionally, it has been extensively demonstrated that these skills are strongly affected by drug misuse [25]. However, it is not clear whether cognitive and empathic impairments might be due to drug misuse or vice versa, although it is clear that drug misuse exacerbates these deficits. Hence, it makes sense that these variables might play an important role in modulating low treatment adherence, dropout, and IPVAW recidivism.

Regarding these empathic and cognitive abilities, empathy has usually been divided into cognitive and empathic components. Cognitive empathy has been defined as the ability to understand how someone else sees the world, and the ability to consciously enter the mind of another person (perspective taking). However, emotional empathy, or the ability to understand or feel what someone else feels and engage in "experience sharing”, should also be considered [19,20,21]. These processes are partly sustained by the ability to decode emotions in facial expressions, which offer a basic source of information with which to infer another individual’s perspective. Finally, empathic abilities are interrelated with executive functioning. An important process is a cognitive flexibility or the ability to avoid using rigid cognitive schemas or behavioural patterns when facing a demanding and variable context. All these abilities are partly involved in moral reasoning, prosocial behaviour, social and emotional adequacy, mood, and behaviour regulation [26,27]. Therefore, diminished functioning of these abilities might entail difficulties in correctly regulating emotions and behaviours.

Deficits in these basic empathic and cognitive processes might explain impulsive and non-reflective decision-making, such as abandoning the treatment during initial stages without considering the future consequences of this decision. This explanation is consistent with the ‘somatic marker hypothesis’ proposed by Damasio, Tranel, and Damasio [28]. This hypothesis proposes that individuals characterized by impulsive or non-reflective decisions tend to fail to use the information available in the environment to foresee the consequences of their behaviour. Therefore, it seems logical that alterations in these abilities might make IPVAW perpetrators with important empathic and cognitive impairments feel overwhelmed by the theoretical and emotional context of the intervention and, thus, drop out, without considering the future effects of their decision. Therefore, it is necessary to analyse these variables because treating them could reduce the risk of reoffending by influencing treatment engagement in the initial stages.

In accordance with these results, the primary objective of the present prospective study was to compare empathic abilities (cognitive and emotional), emotion decoding abilities cognitive flexibility, the initial risk, and the final recidivism, based on official records, of a carefully selected sample of IPVAW perpetrators who completed the intervention (*n* = 360; mean age = 41) and a group of participants who dropped out (*n* = 93; mean age = 39). Furthermore, these two groups were divided into two additional groups depending on whether or not they presented alcohol misuse. Based on previous research in this field that demonstrated the existence of diminished empathic and cognitive skills in IPVAW with alcohol misuse [22,23,24,29,30,31,32] and a higher dropout rate in IPVAW offenders with high alcohol misuse [8], our hypothesis was that the worst empathic and cognitive abilities and the highest recidivism rate would be presented by IPVAW perpetrators with treatment noncompliance and alcohol misuse. Conversely, IPVAW offenders who completed the intervention, especially those with low alcohol use, would present greater empathic and cognitive flexibility than those who discontinued the intervention, as well as a lower risk and final rate of IPVAW recidivism.

## 2. Methods

### 2.1. Participants

Participants for this study were recruited from January 2013 to December 2018. From an initial sample of 471 IPVAW perpetrators who agreed to participate, only 423 were included in the study because they completed all the measures of interest for this study. The final sample was divided into four groups: those who completed the intervention with low and high alcohol consumption (*n* = 267 and *n* = 67, respectively) and those who dropped out before the intervention ended with low and high alcohol consumption (*n* = 62 and *n* = 27, respectively). IPVAW perpetrators received a court-mandated psycho-educational and community-based treatment program (CONTEXTO) at the University of Valencia (Valencia, Spain). This intervention program receives approximately 120 IPVAW perpetrators every year. There are several CBT-oriented BIPs in Spain that includes topics such as sexism, sex roles, and gender equality. The CONTEXTO program is not only based on CBT, but it also includes motivational strategies in order to increase compliance with the treatment and motivation for change [5]. Since they were sentenced to less than two years in prison, with sentences ranging from 6 to 24 months, and had no previous criminal record, participants had been given a suspended sentence on the condition that they would attend an intervention program [5]. In fact, in the CONTEXTO program, only men over 18 years of age with no physical or severe mental/cognitive problems (e.g., schizophrenia, severe traumatic brain injury, strokes with severe brain dame...) were included. Moreover, they could not present severe substance use disorders, which can produce disruptive behaviours in intervention programs. All of them agreed to participate voluntarily in the study, previously signing the informed consent. The study followed the Declaration of Helsinki, and it was also approved by the University of Valencia Ethics Committee, with the following assigned code: H1348835571691.

### 2.2. Procedure

Before agreeing to participate, all the IPVAW perpetrators were initially informed that refusal to participate in the study would not affect their legal status. Moreover, all the measurements and answers provided during the interviews would be confidential, and the judicial system would not have access to them. The data were collected before the IPVAW intervention started, as part of the initial assessment in the CONTEXTO program [3,8,33], except for recidivism data (i.e., assessed by therapists and based on official records), which were collected during the last sessions and the first year after the intervention program ended, respectively. The majority of instruments included in this study were self-reports (i.e., audit, Plutchik’s Impulsivity Scale, and the interpersonal reactivity index) and neuropsychological tests (i.e., eyes test and Wisconsin card sorting test). We chose these instruments due to their reliability and validity properties. Moreover, all of them have been extensively employed with violent populations [4,5,6,7,8,9,10,11,12,13,14,15,16,17,18,19,20,21,22,23,24,25,26,27].

Each subject participated in two sessions in the faculty of psychology at the University of Valencia. In the first session, IPVAW perpetrators were interviewed to exclude any individuals with physical or mental illnesses that could seriously disrupt the functioning of the intervention. Moreover, anthropometric and sociodemographic data were collected. The second session took place the following day between 10 a.m. and 2 p.m., in order to minimize possible effects of fatigue later in the day. Each session lasted approximately 90 minutes, and the sessions were conducted by four researchers with a practical background in neuropsychological assessment. After arriving at the laboratory, participants were taken to a room where the neuropsychological tests and self-reports were administered.

### 2.3. Alcohol Misuse

The Spanish version of the Alcohol Use Disorders Identification Test (AUDIT) is a self-reported 10-item measure of alcohol consumption. Scores ranged from 0 (never) to 4 (daily or almost daily) [34]. It was designed to detect harmful alcohol consumption, as well as possible dependence. The cut-off score is equal to or greater than 8, which is considered harmful alcohol consumption. Cronbach’s alpha for this study was 0.77.

### 2.4. Self-Reports and Neuropsychological Tests

#### 2.4.1. Impulsivity Assessment

Plutchik’s Impulsivity Scale is a self-report [35] employed to assess impulsivity as an immediate reaction, disregarding any behavioural consequences. It is a Likert-type scale with a four-point response range (1 = never; 4 = almost always). Cronbach’s alpha was 0.72.

#### 2.4.2. Empathy Assessment

The Eyes Test was developed by Baron-Cohen, Wheelwright, Hill, Raste, and Plumb [36]. This test measures emotion-decoding abilities by identifying the emotion that best describes this part of the facial expression in 36 black and white photographs of both genders. The total score is obtained by adding up the number of correct answers, ranging from 0 (poor ability) to 36 points (stronger emotion-decoding abilities).

The Spanish version of the interpersonal reactivity index (IRI) [37] was employed to measure cognitive (perspective taking) and emotional empathy (empathic concern and personal distress). Its items were rated from 1 (does not describe me well) to 5 (describes me well). Cronbach’s alpha for this study was 0.79.

#### 2.4.3. Executive Function (Cognitive Flexibility)

The Wisconsin Card Sorting Test (WCST) measures abstract reasoning and the ability to change cognitive strategies based on feedback from the administrator. It consists of four stimulus cards and 128 response cards containing various colours (red, blue, yellow, or green), shapes (circle, cross, star, or triangle), and numbers (one, two, three, or four) of figures [38]. Participants have to match the response cards to one of the stimulus cards. Dependent scores in this study were total errors, perseverative errors, and completed categories.

#### 2.4.4. Recidivism/Reoffending Risk

The Spanish version of the Spousal Assault Risk Assessment (SARA) was used to measure the recidivism risk [39]. This assessment is a 20-item protocol in the form of a clinical checklist that includes the main risk factors for IPVAW. Responses range from 0 (not present) to 2 (present). Higher scores indicate higher recidivism risk.

### 2.5. Recidivism/Reoffending Rate

The rate of reoffending was assessed for one year after the treatment ended, by the monitoring system of the Spanish Ministry of the Interior (responsible for the penitentiary system). Recidivism was registered as an IPVAW incident, other types of criminal and/or violent activities, or a breach of the conditions mandated by a judge. We coded this variable as 0 (if the participant did not reoffend) and 1 (if he reoffended).

### 2.6. Data Analysis

After using the Kolmogorov-Smirnov to test whether the majority of the data followed the normal distribution (*p* < 0.05), we decided to perform the Kruskal-Wallis test, including ‘group’ (four groups including dropout and alcohol misuse) as the between-subject factor for the anthropometric, psychological, and neuropsychological data. The post hoc analysis was performed using the Mann-Whitney test. Moreover, chi-square statistics were calculated to analyse the frequencies of the demographic variables.

Pearson or Spearman correlation coefficients were calculated to assess relationships between recidivism (SARA and official recidivism) and self-reports and neuropsychological tests for each group.

Data analyses were carried out using IBM SPSS Statistics for Windows, version 24.0 (IBM Corperation, Armonk, NY, USA). Results were considered statistically significant with *p* values ≤ 0.05.

## 3. Results

Table 1 presents descriptive characteristics for the IPVAW perpetrators who dropped out of the intervention and those who completed it. The four groups were similar on all the socio-demographic variables. However, there were differences between groups in the number of treatment sessions received, the initial risk of IPVAW recidivism assessed by the SARA, and the rates of reoffending assessed by the Spanish Home Office and collected one year after treatment ended. The post hoc analysis revealed that the IPVAW who completed the intervention with low alcohol consumption received a higher number of sessions and presented less initial risk of reoffending and real reoffending than the rest of the groups (*p* < 0.05), particularly less than those who dropped out with high alcohol misuse (*p* < 0.001).

### 3.1. Self-Reports and Neuropsychological Assessment

With regards to self-reported impulsivity, differences were found between groups, Kruskal-Wallis chi-squared = 11.91, *p* = 0.008, with both groups of IPVAW perpetrators with low alcohol consumption presenting lower self-reported impulsivity than those with alcohol misuse (*p* < 0.01 for all).

With regard to empathic variables (perspective taking, empathic concern, and personal distress) and the eyes test, significant group differences were found on the eyes test, Kruskal-Wallis chi-squared = 9.33, *p* = 0.009, with both groups of IPVAW who completed the intervention presenting higher scores than those who dropped out and presented high alcohol misuse (*p* < 0.001 and 0.005, respectively). Finally, IPVAW perpetrators who dropped out of the intervention and had low alcohol consumption presented a higher score on the Eyes Test than those with high alcohol consumption (*p* = 0.005).

Regarding WCST performance, differences were found between groups on the total number of errors, Kruskal-Wallis chi-squared = 8.88, *p* = 0.012, and perseverative errors, Kruskal-Wallis chi-squared = 9.52, *p* = 0.009. The post hoc analysis revealed that both groups of IPVAW offenders who completed the intervention (low and high alcohol misuse) presented fewer errors than both groups of IPVAW perpetrators who dropped out of the intervention (*p* < 0.01 for all). Moreover, both groups of IPVAW who completed the intervention presented fewer perseverative errors than those who discontinued the program and had high alcohol misuse (*p* < 0.001 for all). Finally, IPVAW perpetrators who dropped out of the intervention and had low alcohol consumption presented fewer perseverative errors than those with high alcohol consumption (*p* = 0.049).

### 3.2. Relationship of Recidivism (SARA and Official Recidivism) with Self-Reports and Neuropsychological Tests for Each Group

SARA (risk of recidivism assessed by therapists) was significantly and positively associated with self-reported impulsivity in those IPVAW perpetrators who completed the intervention and those who dropped out, but with low alcohol use (r =−0.275, and r = 0.402, *p* < 0.01, respectively). Moreover, SARA was also negatively associated with IRI empathic concern (r = −0.125, *p* < 0.05) in those IPVAW perpetrators who completed the intervention and presented low levels of alcohol use.

Regarding official reoffending, there was only a significant and negative association with IRI perspective taking in IPVAW perpetrators who dropped out with low and high levels of alcohol use (r = −0.305, and r = −0.409, *p* < 0.05, respectively).

## 4. Discussion

The present study demonstrated that IPVAW perpetrators who did not complete the intervention, particularly those with high alcohol use, were less accurate in decoding emotional facial signals, and presented higher total and perseverative errors than those who completed the intervention, especially in comparison with those with low alcohol consumption. Furthermore, they also presented the highest risk of recidivism and official reoffending rates (assessed by official records) in the first year after the intervention ended.

The main objective of the current study was to assess whether there were differences in the neuropsychological performance and reoffending rates of groups of IPVAW offenders who completed or did not complete the intervention, while also considering their alcohol use patterns (low vs. high). It should be noted that, in general, the findings partly show that IPVAW offenders with alcohol misuse presented worse empathic and cognitive performance than those with low alcohol consumption. We failed to find differences between IPVAW perpetrators who completed the program with different alcohol patterns, but we found differences between non-completers with and without alcohol misuse. In fact, non-completers with low alcohol consumption performed better than those characterized by alcohol misuse. Differences were found especially between both groups of completers and the groups that did not complete the intervention. Moreover, it is particularly interesting that high self-reported impulsivity and low empathic scores (perspective taking and empathic concern) were associated with high reoffending rates in IPVAW perpetrators.

If we try to explain the lack of differences among IPVAW completers based exclusively on their alcohol misuse or self-reported impulsivity, it should be clarified that the AUDIT and Plutchik scores of both groups of alcohol misusers were similar. Hence, it would be necessary to employ other hypotheses that could help to explain the absence of differences. It is possible that non-completers with alcohol misuse presented a longer history of alcohol misuse, abuse of multiple drugs, or different periods of abstinence, among others. Therefore, these factors related to drug misuse should be considered in future research in order to determine different profiles of IPVAW perpetrators. Moreover, recent studies demonstrated that a large percentage of IPVAW perpetrators with heavy alcohol consumption over a long period of time, tend to present deficits in sustained and divided attention and working memory [40,41]. These processes support other higher-order cognitive processes, such as emotion decoding and cognitive flexibility [42,43]. Thus, the absence of differences among completers and the presence of differences in non-completers might be explained by attention and working memory impairments that sustain or are interrelated with higher-order cognitive processes (e.g., empathy, executive functioning). Thus, future studies should assess these variables (i.e., multiple drug use and a complete neuropsychological assessment).

It is particularly interesting that, as expected, the highest reoffending rate was shown by non-completers with alcohol misuse, and, conversely, the lowest rate was shown by completers with low alcohol consumption, which is consistent with previous models established in this line of research [3,4,5,6,7,8,9]. This result also reinforces the need to reduce alcohol consumption directly through interventions that should be parallel to IPVAW treatment or considered part of treatment packages. Lastly, the lowest rate of reoffending corresponded to the best neuropsychological performance, and the highest rate corresponded to the worst performance. It was previously established that the CONTEXTO program is a valid intervention to promote executive functioning (i.e., cognitive flexibility, decision-making process…) and empathic improvements in IPVAW perpetrators who completed the program. In this regard, it was demonstrated that training in cognitive restructuring and emotion management skills, which is part of the intervention program, would partly explain these improvements. However, further studies are necessary to develop coadjutant interventions that help to produce greater improvements [30]. These results offer a target for future interventions in designing intervention packages that focus on reinforcing treatment adherence in the first stages of the intervention. Additionally, several studies have demonstrated that emotion decoding and cognitive flexibility have improved through training in several non-violent populations [44,45,46] and in IPVAW perpetrators [20,21,22,23,24,25,26,27,28,29,30].

Despite the strengths of our study, several limitations should be considered. First, this is a prospective cohort study that only assessed participants one year after the treatment ended. Hence, it is possible that certain individuals reoffended after this period of time. Moreover, the official records provided by a legal institution did not specify the type of reoffence (i.e., physical, psychological, sexual…), which was only based on a dichotomous response (e.g., presence/absence). Second, the assessment of alcohol misuse was based on a self-report, without considering the amount of daily alcohol consumed, the number of years with sustained heavy alcohol consumption, the presence or not of an alcohol use disorder (AUD), multiple drug consumption, among others, which are relevant in cognitive decline [41]. Third, the use of self-reports is subject to social desirability and/or cognitive distortion [47]. Fourth, although neuropsychological tests are not easily manipulated, there are factors that were not considered in our study that might easily distort neuropsychological performance, such as sleep quality, craving in participants with AUD, the current level of stress due to their legal situation, etc. Furthermore, our results could be reinforced by other qualitative and quantitative parameters related to the intervention program (i.e., behavioural control during therapy, personality and emotional assessment, or the therapeutic alliance [48]). Fifth, we only focused on IPVAW perpetrators around Valencia. Thus, future studies should consider not only Spanish IPVAW perpetrators but also perpetrators from other countries, in order to increase the external validity of our study. Finally, to obtain recidivism rates, we considered two measurements, therapist reports and official reports, without considering the information provided by victims. Nonetheless, Spanish legislation does not allow us to directly contact victims, in order to avoid any type of harm (i.e., psychological).

## 5. Conclusions

In summary, this study shows that IPVAW perpetrators who do not complete the treatment and misuse alcohol are more likely to reoffend. However, IPVAW treatment completers without alcohol misuse show the lowest rate, followed by completers with alcohol misuse. In this regard, the present study highlights the need to design more effective adherence strategies for IPV perpetrators, especially those with alcohol misuse, based on strategies targeting cognitive deficits, drug misuse, or both.

## Figures and Tables

**Table 1:** Mean ± SD of descriptive characteristics for all groups (* *p* < 0.05, ** *p* < 0.01, *** *p* < 0.001).

	No Dropout (*n* = 334)		Dropout (*n* = 89)	
	Low Alcohol (*n* = 267)	High Alcohol (*n* = 67)	Low Alcohol (*n* = 62)	High Alcohol (*n* = 27)
**Age (years)**	40.46 ± 11.69	40.13 ± 10.77	40.23 ± 13.27	35.89 ± 7.78
**Nationality**				
Spanish	76%	76%	76%	67%
Other nationalities	24%	24%	24%	33%
**Marital status**				
Married	23%	27%	26%	22%
Single/Separate/Divorced/Widowed	77%	73%	74%	78%
**Level of education**				
Primary/lower secondary	54%	61%	56%	63%
Upper secondary/vocational training	46%	39%	44%	37%
**Employment status**				
Employed	57%	48%	40%	53%
Unemployed	43%	52%	60%	47%
AUDIT scores ***	2.39 ± 2.34	12.79 ± 5.29	3.39 ± 2.81	13.07 ± 4.88
Self-reported impulsivity (Plutchik) **	31.59 ± 5.22	33.76 ± 5.77	31.23 ± 6.13	32.74 ± 4.55
Number of treatment sessions ***	28.60 ± 5.74	28.24 ± 5.86	10.68 ± 7.92	10.29 ± 9.34
**Physical violence against women**				
Yes	69%	69%	68%	54%
No	31%	31%	32%	46%
SARA ***	8.55 ± 4.89	11.63 ± 5.66	11.68 ± 6.13	13.50 ± 5.85
IPV recidivism (VIOGEN database) ***				
Yes	8%	18%	27%	30%
No	92%	82%	73%	70%
**Self-report and Neuropsychological tests (empathy, emotion decoding abilities, and cognitive flexibility)**				
IRI Perspective taking	21.39 ± 4.77	21.05 ± 4.69	19.82 ± 5.33	20.33 ± 4.79
IRI Empathic concern	21.13 ± 4.15	22.57 ± 4.47	21.15 ± 4.89	21.59 ± 4.22
IRI Personal distress	17.06 ± 4.04	18.74 ± 4.22	16.95 ± 4.30	18.67 ± 4.65
Eyes test **	18.40 ± 4.17	18.15 ± 4.28	18.11 ± 4.20	15.29 ± 5.23
WCST				
Number of errors **	49.35 ± 25.33	47.58 ± 25.60	59.48 ± 23.86	62.07 ± 23.03
Number of perseverative errors **	26.16 ± 17.53	25.12 ± 15.93	29.43 ± 17.58	39.67 ± 22.88
Number of categories completed	2.59 ± 1.49	2.69 ± 1.54	3.06 ± 1.44	2.78 ± 1.69
